# Ediacaran skeletal metazoan interpreted as a lophophorate

**DOI:** 10.1098/rspb.2015.1860

**Published:** 2015-11-07

**Authors:** A. Yu. Zhuravlev, R. A. Wood, A. M. Penny

**Affiliations:** 1 Department of Biological Evolution, Faculty of Biology, Lomonosov Moscow State University, Leninskie Gory, Moscow 119991, Russia; 2 School of GeoSciences, University of Edinburgh, West Mains Road, Edinburgh EH9 3FE, UK

**Keywords:** Ediacaran, Cambrian, lophophorate

## Abstract

While many skeletal biomineralized genera are described from Ediacaran (635–541 million years ago, Ma) strata, none have been suggested to have an affinity above the Porifera–Cnidaria metazoan grade. Here, we reinterpret the widespread terminal Ediacaran (approx. 550–541 Ma) sessile goblet-shaped *Namacalathus* as a triploblastic eumetazoan. *Namacalathus* has a stalked cup with radially symmetrical cross section, multiple lateral lumens and a central opening. We show that the skeleton of *Namacalathus* is composed of a calcareous foliated ultrastructure displaying regular concordant columnar inflections, with a possible inner organic-rich layer. These features point to an accretionary growth style of the skeleton and an affinity with the Lophotrochozoa, more specifically within the Lophophorata (Brachiopoda and Bryozoa). Additionally, we present evidence for asexual reproduction as expressed by regular budding in a bilateral pattern. The interpretation of *Namacalathus* as an Ediacaran total group lophophorate is consistent with an early radiation of the Lophophorata, as known early Cambrian representatives were sessile, mostly stalked forms, and in addition, the oldest known calcareous Brachiopoda (early Cambrian Obolellida) and Bryozoa (Ordovician Stenolaemata) possessed foliated ultrastructures.

## 1. Introduction

The Cambrian radiation (starting at approx. 541 Ma) records the seemingly abrupt appearance of biomineralized animals with highly sophisticated skeletons in the geological record. These are represented by calcareous and phosphatic shelly fossils of triploblastic bilaterians, including various molluscs, brachiopods and diverse stem group lophotrochozoans in the early Cambrian Terreneuvian Epoch (approx. 541 to approx. 525 Ma) followed by biomineralized arthropods, other ecdysozoans and echinoderms during the Cambrian Epoch 2 [1,2].

Over 10 genera with biomineralized skeletons are known from the terminal Ediacaran (approx. 550–541 Ma) [3–7]. Most have been interpreted to be either protistan *sensu lato* or poriferan–cnidarian grades of organization on the basis of overall morphology and the presence of simple skeletal ultrastructures of microgranular or microfibrous types [8,9].

Of these, *Namacalathus* is found in carbonate strata from the Nama Group, Namibia [4,10], the Byng Formation of the Canadian Rocky Mountains [11], the Birba Formation of Oman [1,12], the Kolodzha and Raiga formations of West Siberia [13] and the Anastas'ino Formation of the Altay-Sayan Foldbelt [14]. *Namacalathus* has been proposed to represent either a possible cnidarian on the basis of an overall goblet-shaped morphology and hexaradial cross section [4], a protozoan owing to small size and an apparent lack of accretionary growth [15], a stem-eumetazoan [16] or as a lophotrochozoan based on a microlamellar ultrastructure as manifest in limited petrographic thin section material [6].

Here, we document additional features of growth form and skeletal structure from exceptionally well-preserved and abundant material of the widespread species *Namacalathus hermanastes* from the terminal Ediacaran (approx. 548–541 Ma) of the Nama Group, Namibia. This allows some potential constraints to be placed on original skeletal mineralogy, soft-tissue distribution and mode of biomineralization, as well as insights into the possible affinity of *Namacalathus*.

## 2. *Namacalathus* general morphology

**Figure 1.**
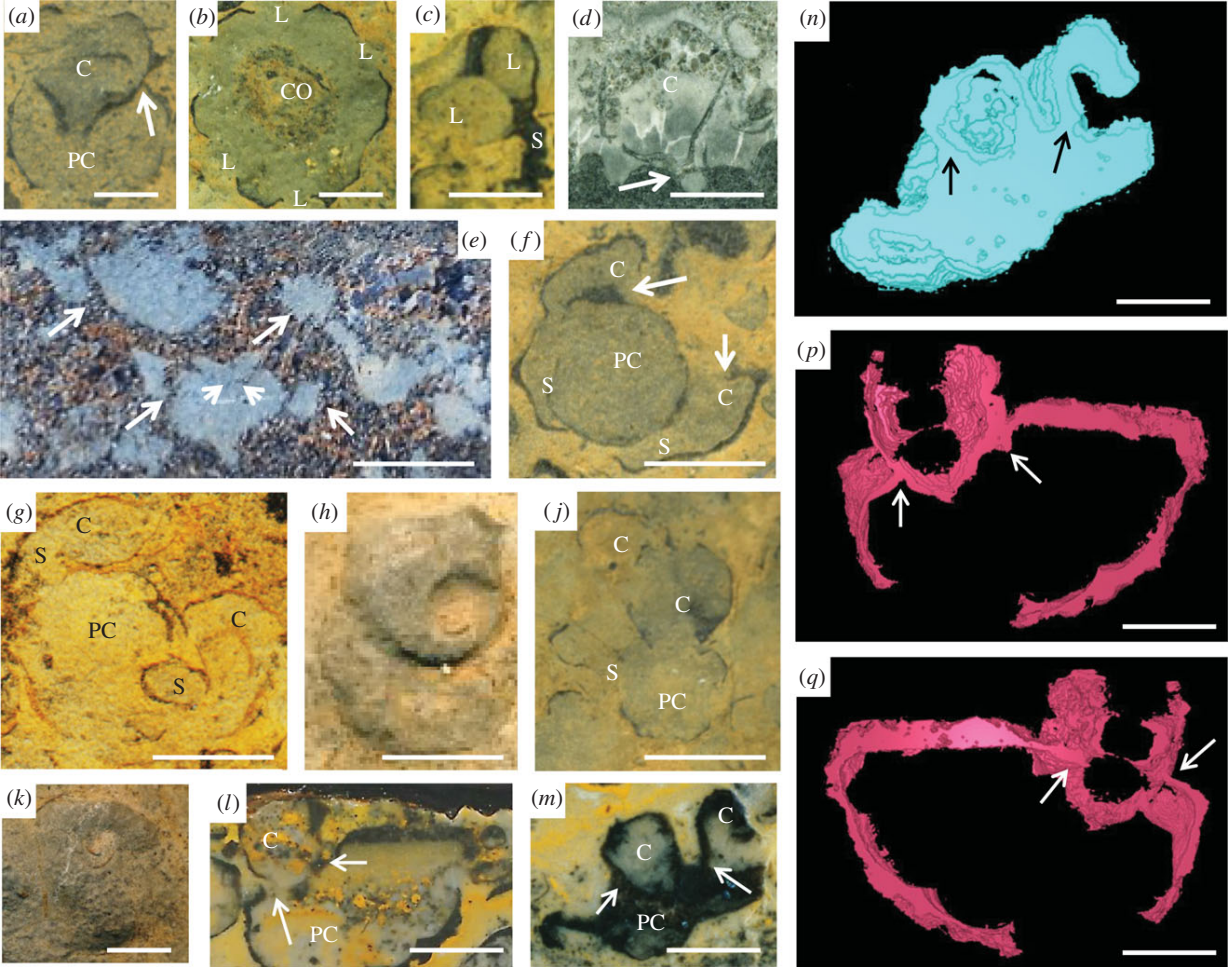
*Namacalathus hermanastes* from the Nama Group, Namibia. Scale bars, 5 mm. (*a*) Longitudinal section through individual shows parental cup (PC) and daughter cup (C) with continuous skeletal wall (arrowed). (*b*) Transverse section through individual cup with hexaradial symmetry and lumens (L) and central opening (CO). (*c*) Longitudinal section through single individual showing lumens (L) and stem (S). (*d*) Individual enclosed in micritic sediment and attached to a microbial mat shows a bulb-like initial shell with constriction at the junction with the main skeleton (arrowed). (*e*) Longitudinal section of closely aggregated individuals with daughter individuals budding from older ones (arrowed). (*f,g*) Transverse section showing parent cup (PC) with two and budded daughter cups (arrowed) which initiate as stems (S) then inflate to cups (C). (*h,k*) Transverse section shows spiral form as continued growth the new cup stems curved around the axis of the parent cup. (*j*) Possible third generation of daughter cups (C), budding from parental cup (PC) and stem (S). (*l,m*) Longitudinal sections through individual showing parental cup (PC) and daughter cup (C) with continuous skeletal wall (arrowed). (*n,p,q*) Three-dimensional model reconstructions from serial sections (see electronic supplementary material). (*n*) Based on sections from sample in *m, (p,q*), based on sections from sample in L, with arrows in same positions.

*Namacalathus* has a stalked cup- or goblet-shaped form up to 35 mm in width and height, with a stem serving as attachment to a substrate and a cup possessing a rounded central opening on the top and usually six, but sometimes, five or seven further rounded lumens on lateral facets [4,17] (figure 1*c*). *Namacalathus* displays an almost regular hexagonal radial symmetry in cross section [4,17] (figure 1*b*), although five and sevenfold radial cups based on the number of lateral facets also occur. The stem and outer cup surface can be covered with short, robust spines which previously have been identified in *Namacalathus* from the Ediacaran Byng Formation of the Canadian Rocky Mountains only [11]. The wall of both the stem and the cup is continuous (figure 1*a*,*l,m*) and up to 100 µm thickness.

We have noted in two longitudinal sections through two specimens that the distal, lowermost, part of the stem is closed. In the cases of *in situ* attached individuals, the lowermost stem part is separated from the remainder of the skeleton by a transverse septum-like structure resulting in a bulb-like, initial shell appearance (figure 1*d*).

## 3. Skeletal ultrastructure

The skeleton of *Namacalathus* has been variously interpreted as either calcite [4] or high-magnesium (Mg) calcite [18] on the basis of an absence of neomorphic calcitic textures or moldic preservation, but no detailed study of ultrastructure has been documented.

**Figure 2.**
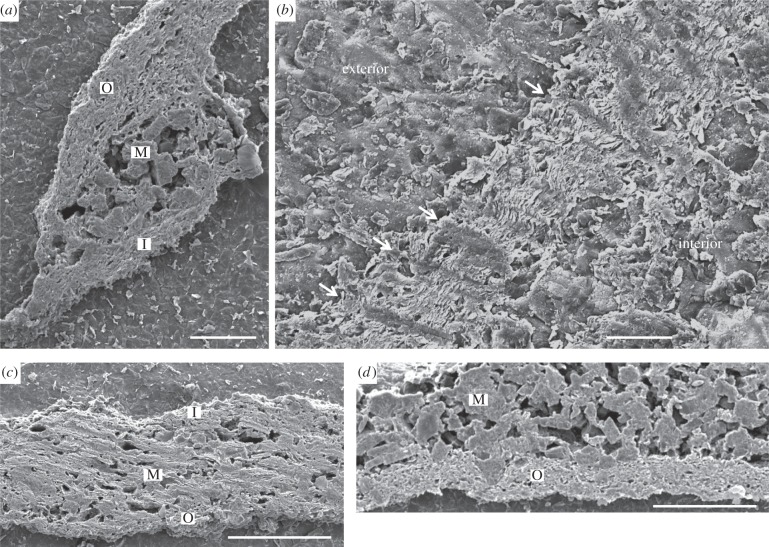
Secondary emission SEM images of etched and polished transverse sections of *Namacalathus hermanastes* skeletal wall ultrastructure from the Nama Group, Namibia. (*a,c,d*), Tripartite organization. M, internal (middle) layer of rod-like microdolomite crystals; O, external outer foliated layers. I, inner foliated layers. (*a*) Scale bar, 200 µm. (*c*) Scale bar, 100 µm. (*d*) Scale bar, 100 µm. (*b*) Columnar microlamellar inflections (arrowed). Orientation with respect to the interior and exterior of the cup is noted. Scale bar, 200 µm.

**Figure 3.**
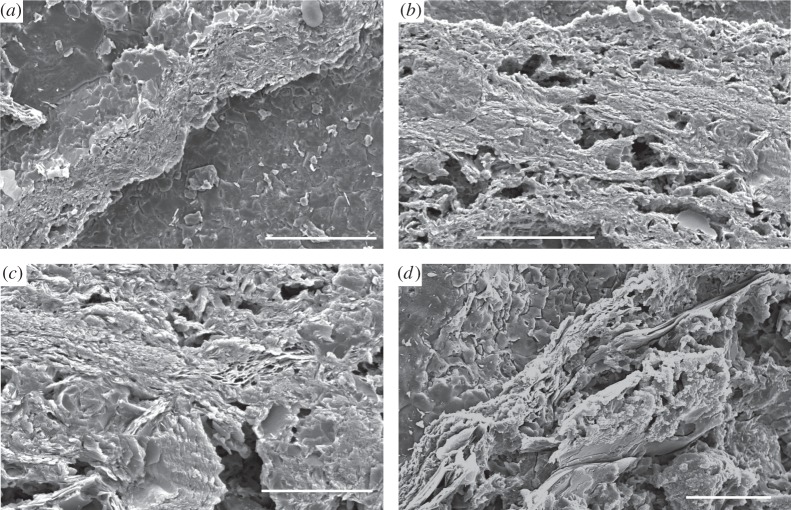
Secondary emission SEM images of etched and polished transverse sections of *Namacalathus hermanastes* external, outer skeletal wall ultrastructure from the Nama Group, Namibia, shows regular foliated ultrastructure with closely spaced microlaminae. (*a,b*) Scale bars, 100 µm. (*c,d*) Scale bars, 50 µm.

Scanning electron microscope (SEM) imaging reveals both the cup and stem parts of the *Namacalathus* skeleton to present a tripartite structure of two thin external (outer and inner) laminar layers enclosing an internal space (middle layer) infilled by irregularly freely arranged rod-like crystals (figure 2*a*,*b,d*). The outer and inner laminar layers show a platy structure of individual microlaminae in an ultrathin petrographic section [6]. These laminated skeletal layers have a fine-scale ultrastructure of multiple, closely spaced, continuous, regularly foliated microlaminae of approximately 1.25 µm in thickness each in average (figure 3). Microlaminae are oriented parallel to the skeletal surface and follow its undulations and inflections (figure 3*a*,*c*). Abundant rod-like microdolomite crystals are either restricted to the middle layer (figure 2*a,d*) or concentrated along the outer surface.

Commonly, microlaminae become denser to form arcuate, inclined trails of tight columnar cone-in-cone deflections in the skeleton lamination orientated normal to the skeletal surface and crossing the entire laminar layer (figure 2*b*). These deflections are spaced 10–30 µm apart and point towards the inside of the skeleton.

## 4. Taphonomic inference from sediment infill

The sediment infill of *Namacalathus cups* often differs in colour and/or grain size from externally adjacent sediments [4]. The boundary between these two sediment types is as distinct across lateral lumens as it is across solid walls, suggesting the presence of a barrier within the lumens which prevented sediment ingress even some time after the death of the organism (figure 1*b*,*e,f*). By contrast, internal and external sediments are mixed adjacent to the central opening at the top of the cup suggesting that this was open at time of burial [4] (figure 1*b*).

## 5. Potential asexual reproduction

The common close packing of *Namacalathus* individuals on bedding planes infers growth by a regular larval recruitment and settlement [19].

We note, additionally, that some individuals of *Namacalathus* possess daughter cups communicating with a presumable parental cup via stems with inner cavities that are connected directly to the parental cup inner cavity by an orifice (figure 1*e–g*,*j*,*l*,*m*) while skeletal walls are shared [19] (figure 1*a*,*l,m*) and sometimes thickened between new cups and the older one (figure 1*f*,*l*,*m*). Three-dimensional model reconstructions using SPIERS software from serial sections (see electronic supplementary material, figures S1–S4) confirms the skeletal continuity and budding nature of parent and daughter cups (figure 1*n*,*p,q*).

Such an asexual clonal reproduction is comparable to internal budding. Noteworthy is that two new stems of the same diameter and length are attached to opposite outer surfaces of the upper part of a parental cup (figure 1*e*–*g*). This imparts a consistent bilateral symmetry to the entire clonal aggregation. The position of daughter individuals was initially bilaterally symmetrical (figure 1*e*–*g*), but with continued growth, the new cup stems curved around the axis of the parental cup (figure 1*f*,*h,k*). Possible second generations of buds forming cups are observed in some specimens (figure 1*j*).

## 6. Discussion

In the following, we discuss inferences as to original skeletal mineralogy and functional morphology, clonal budding, soft-tissue reconstruction and finally, the possible affinity of *Namacalathus*.

### (a) Diagenesis and original mineralogy

The regular foliated ultrastructure of *Namacalathus* differs drastically from those of co-occurring tubicolous *Cloudina* and the massive honeycomb or calicular growth form of *Namapoikia*. *Cloudina* from the Nama Group possesses a microgranular ultrastructure [6,9], whereas *Namapoikia* displays a coarse calcitic mosaic and its skeleton is commonly overgrown by neomorphosed botryoids of acicular crystals [5]. Co-occurrence of these three ultrastructural varieties removed the possibility of convergent diagenetic pathways implying a primary origin for any ultrastructural differences. Recognition of primary skeletal mineralogies can be assessed following the criteria of James & Klappa [18,20].

A sparry calcite replacement mosaic that generally cross-cuts skeletal structures together with the presence of botryoids typical of primary aragonite marine cement that develops in optical continuity with skeletal elements is indicative of an original aragonite skeletal mineralogy for *Namapoikia*. By contrast, both *Cloudina* and *Namacalathus* skeletons are overgrown by bladed and fibrous cements, and commonly show the growth of microdolomite crystals upon skeletal surfaces. We note that the rod-like microdolomite crystals are restricted to the skeletal middle layer or concentrated along the outer surface of *Namacalathus,* suggesting that the skeleton served as a source of magnesium. These features point to a primary high-Mg calcite mineralogy and preservation of primary skeletal fabrics [20]. We infer the irregular microdolomite rhombohedra within the resultant middle cavity to represent diagenetic precipitates after the post-mortem decay of the organic material enriched by magnesium and seeded by microdolomite crystals.

While a microgranular ultrastructure can originate diagenetically after irregular, spherulitic, fascicular and orthogonal primary fabrics of fibrous type [21,22], a regular foliated ultrastructure has never been observed to be a result of diagenetic alternation of a different primary fabric.

### (b) Skeletal ultrastructure functional morphology

The columnar microlamellar inflections, an organic-rich inner layer and robust external wall spines may all have imparted mechanical strength fracture resistance to *Namacalathus.* Columnar microlamellar inflections are considered to retard the propagation of cracks through a skeleton displaying increasing curvature of wall, and impart resistance to both high-energy currents and predatory attack [23,24].

In molluscan shells, organic-rich layers have also been interpreted to aid the dissipation of energy from hydrodynamic pressure or predation [25]. The organic-rich skeleton with fine microlaminae separated by an organic envelope would also have maintained flexibility and enabled growth in energetic settings. *Namacalathus* was indeed able to occupy energetic and current-swept reefal environments [4,19,26], and skeletal flexibility is apparent by the common preservation of folded or plastically distorted skeletal walls [4].

Early representatives of tommotiids, brachiopods and bryozoans were able to secrete intercrystalline structural organic compounds that penetrated the entire skeleton and were anchored to the epithelium [27–29]. Thus, cone-in-cone deflections may reflect traces of an original sensory organ that would be particularly advantageous for sessile animals which possessed a mineralized exoskeleton that prevented sensory evaluation of the environment through the epithelium.

### (c) Clonal budding

The regular pattern of coexisting and attached individuals of *Namacalathus* is interpreted to be a feature of a clonal development, namely internal budding. During clonal growth, two new potential buds appeared bilaterally symmetrical on the outer surface of the upper part of the inferred parental cup. Such a regular bilaterally symmetrical budding may reflect either the presence of bilaterally spaced reproductive organs or represent a prerequisite for the future development of such organs.

### (d) Soft-tissue reconstruction

The overall morphology of *Namacalathus* is not consistent with a sponge organization as the presence of relatively large lateral lumens of a diameter comparable to that of the central opening would reduce pressure within the filter feeding system by decreasing the initial velocity of the cumulative exhalant jet, in turn leading to permanent recycling of filtered water and potential clogging of choanocyte chambers [30,31].

Formation of a foliated skeletal ultrastructure requires the presence of compound-secreting tissue similar to that of the mantle of some lophophorates or molluscs, but a molluscan suspension-feeding mode would also require the presence of siphon-type organs which is incompatible with the general morphology of *Namacalathus*.

The taphonomic features of sediment ingress within the *Namacalathus* skeleton allow us to infer the presence of some type of non-skeletal tissue within the lumens, but none adjacent to the central opening. Such a soft tissue distribution is not consistent with the presence of multiple tentacle-ringed mouths as in a microcarnivorous sessile colonial cnidarian, but rather with the existence of a suspension-feeding organ within the cup of *Namacalathus*.

### (e) Affinity of *Namacalathus*

To aid interpretation of possible affinity, the characteristics of *Namacalathus* are summarized in table 1 along with those from high-ranking modern taxa (calcareous algae, foraminiferans, sponges, cnidarians and various crown group bilaterians) and some extinct sessile organisms with calcareous and phosphatic skeletons (Ordovician–Jurassic tentaculitoids and early Cambrian tommotiids).

**Table 1.** Distribution of characteristics observed in *Namacalathus* among high-rank taxa of modern and extinct sessile calcareous organisms [27,32–42].

features/taxa	*Namacalathus*	algae	foraminifera	porifera	cnidaria	annelida	mollusca	Brachiopoda	Bryozoa	Tentaculitoidea (Ordovician–Jurassic)	Tommotiida (early Cambrian)
Mg-calcite	X	X	X	X	X	X	egg capsule only	X	X	X	
microlamellar foliated ultrastructure	X						X	X	X	X	X
columnar inflections of laminae	X							X	X	X	
skeleton radial symmetry	X	X	X	X	X	X			X	X	X
stalked skeleton	X	X	X	X	X		X	X	X	X	X
cup with lumens	X	X		X	X				colonial forms		
larval bulb/shell	?						X	X	X	X	X
internal budding	X	Superficially similar features		X	X	X			X	X	
paired buds	X								X	**X**	

We note that many features of *Namacalathus* are widely distributed among unrelated taxa and so offer limited insight into affinity. These include a high-Mg calcite skeletal composition, radial symmetry and a stalked appearance with a cup bearing large lumens. The presence of a bulb-like feature at the base of the *Namacalathus* stem (figure 1*d*) may represent initial growth stages anchored within or attached to microbial mats. Such a bulb-like larval shell showing a distinct constriction at the junction with the main skeleton is observed in some bryozoans and tentaculitoids [27,32]. More material, however, is required to support this initial observation.

The regular bilaterally symmetrical budding may reflect either the presence of bilaterally spaced reproductive organs or represent a prerequisite for the future development of such organs. In clonal lophotrochozoans, including phoronids, entoprocts, microconchids (tentaculitoids), cycliophorans and some bryozoans, buds are similarly developed in the distal, outermost parts of parental individuals and can produce a bilaterally symmetrical pattern of conjoined, paired and in the case of microconchids also coiled, buds [43–46].

A foliated skeletal ultrastructure, columnar microlamellar skeletal deflections and internal budding have a restricted taxonomic distribution (table 1). The open texture of the middle skeletal layer in *Namacalathus* is reminiscent of the interlaminar lenticular chambers found in lingulate brachiopods which contains apatite aggregate meshes [47], and of organic layers in high-Mg calcitic shells of the Craniaformea which bear a high concentration of organic material and magnesium [48,49].

Regular, foliated ultrastructure is restricted only to the accretionary-growing shells of molluscs [50], brachiopods and bryozoans [27]. In addition, tight columnar cone-in-cone deflections within skeletal laminations identical in form and scale to structures to those noted in *Namacalathus* are found in some brachiopod and bryozoan taxa, both of which belong to the Lophophorata [27,33]. In brachiopods and bryozoans such structures may form trabeculae on the inner shell surface and contain rods, where they are known variously as pseudo-punctae or acanthostyles [27,28].

Such features are not found in any other modern skeletal groups, but are also found in the extinct tentaculitoids and hederelloids. These are interpreted as possible extinct biomineralized stem group phoronids representing the third modern branch of the Lophophorata [27,32]. Of particular note is the ‘canalicular’ ultrastructure of early Cambrian obolellid brachiopods which shows fine calcareous laminations and cone-in-cone deflections at the same scale as noted in *Namacalathus* [34,35]. These forms are the first known brachiopods with a calcareous shell and appear in the basal Cambrian Stage 2 [2,18]. Obolellids may, therefore, provide a potential microstructural intermediate between *Namacalathus* and pseudo-punctate brachiopods. Similar linear structures which can deflect shell layers are also common in Cambrian tommotiids, e.g. *Micrina,* which are considered to be stem group brachiopods or lingulate brachiopods [29,35–37], although these are found in inferred primary phosphatic mineralogy. We tentatively conclude that foliated ultrastructure and tight columnar cone-in-cone deflections within the skeletal walls may represent characters limited to the Lophophorata.

Modern calcareous brachiopod shells and the exterior walls of bryozoan skeletons are accreted from a shell-lining secretory epithelium which first secretes an organic layer that acts as a template for biomineralization. The epithelium then changes its secretory regime to form calcareous layers and the resultant calcified skeletal layer is sandwiched between the secretory epithelium and an organic periostracum or cuticle [28,33]. The arcuate trail and inclination of columnar microlamellar inflections are growth effects that result from the mantle pushing radially outwards in response to thickening of the shell layer and marginal expansion of the skeleton [33]. Formation and accretion of a foliated calcitic ultrastructure requires a set of highly heterogeneous organic matrices composed of a large number of different genetically controlled shell proteins that play a critical role in determining the ultrastructure and material properties of the mature biominerals [50,51]; such a set of organic matrices is not observed in prismatic, fibrous or spherulitic ultrastructures which are formed by epitaxial growth from seed crystals within an organic matrix of variable origin [52].

We suggest a similar biomineralization mechanism for the formation of the foliated ultrastructure of *Namacalathus* to that of the Lophophorata. We infer an original mineralogy of high-Mg calcite where the growing skeleton was enveloped similarly to accretionary-growing brachiopod shells and bryozoan skeletons by an organic matrix secreted by a mantle. This may have been combined with a genetic toolkit that encoded for a heterogeneous organic matrix which was capable of forming a complex ultrastructure. *Namacalathus* skeletal microstructure, however, predates the hierarchical composite skeletal architectures typical of more advanced lophotrochozoans.

**Figure 4.**
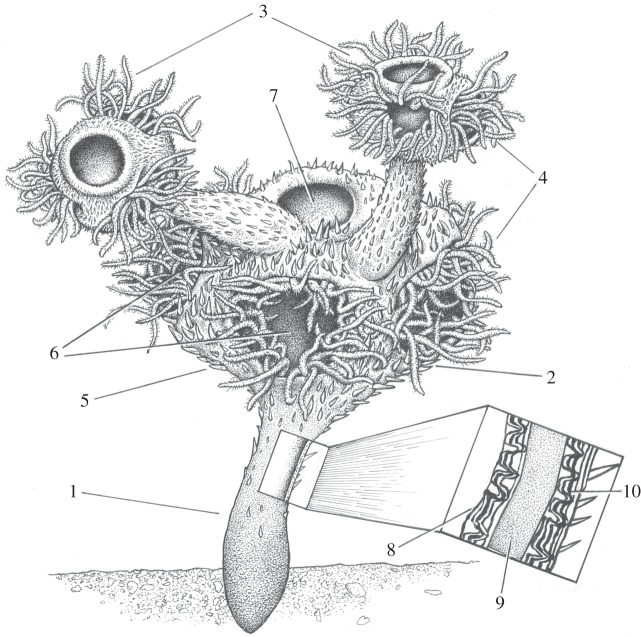
Reconstruction of the living *Namacalathus*. 1, stem; 2, parental cup; 3, daughter cups; 4, hollow ciliated tentacles; 5, spines; 6, lateral lumen; 7 central opening; 8, inner skeletal layer—foliated with columnar microlamellar inflections; 9, internal (middle) skeletal later—organic rich; 10, external outer skeletal layer—foliated with columnar skeletal inflections (image copyright: J. Sibbick).

While there is some debate over precise membership [53,54], there is consensus that the brachiopods, phoronids, annelids, molluscs, bryozoans, entoprocts and some other minor groups form a single clade known as the lophophorates [55]. Modern lophophorates feed on dissolved organic matter and small food particles, especially phytoplankton, being captured before they pass through the field of cilia which creates a feeding current, together with direct absorption of organic compounds via cilia on the tentacles. Such a type of suspension feeding may have been enhanced by the presence of a central opening that acted to generate exhalant upward currents, and the hollow stems would have allowed connection between all asexually budded cups within a colony. This allows a hypothetical reconstruction of *Namacalathus* with ciliated tentacles being protracted from the lateral lumens to form a net of ring-like compartments in order to maximize capture of food particles and/or dissolved organic compounds in regimes of switching current direction (also the inner cup space may have allowed the tentacles to be withdrawn; figure 4). The cilia whipped the particles being captured down into a semicircular food grove connected with a U-shaped gut which ended in an anus at the central opening.

Although the skeleton of *Namacalathus* has hexaradial symmetry, the soft tissue probably did not as the observed bilateral budding supports a bilateral interior organization. We do not suggest that hexaradial symmetry characterized the stem lophophorates. The skeletal and even cuticular organization of many Ediacaran–Cambrian fossils such as Cambrian priapulids and other cycloneuralians [56,57] shows pentameral or hexaradial symmetry. Indeed, even modern priapulids may show a pentameral or hexaradial skeletal organization. There are no known bilateral forms among the extensive array of Ediacaran body fossils, which are either radially symmetrical or asymmetrical. This has led some to suggest a colonial origin for bilaterians from radial ancestors [58].

We note that, in general, early Cambrian lophophorates (brachiopods, tommotiids, the possible entoproct *Cotyledion* and the tentative phoronids *Cambrocornulitus* and *Yuganotheca*) possess skeletons that are either bivalved, sclerital (consisting of small, repeating multiple scale-like elements) or agglutinated [38,59–61]. As a result, some have suggested that ancestral lophophorates bore either a scleritome or a skeleton formed of a lightly mineralized organic-rich cuticle, and that motile lophophorates predated sessile forms [61–63].

The Ediacaran *Namacalathus*, however, possessed a well-mineralized organic-rich skeleton and was sessile. For an early lophophorate to have had such a morphology has support from both molecular and morphological analyses. Molecular phylogenetics suggests that the ancestor of the Entoprocta and the Cycliophora can probably be interpreted as a solitary, marine animal possessing a well-pronounced ability for asexual reproduction [64]. A sister group relationship between the Lophophorata and the Entoprocta and Cycliophora despite the functional differences between the lophophore and the tentacular apparatus of entoprocts and cycliophorans is moderately supported by molecular analysis. This implies that tentacular apparatus for suspension feeding is an adaptation to a sessile lifestyle and is a synapomorphy among these clades [55]. Some cladistic interpretations of brachiopod morphology assume that either the phosphatic Lingiliformea are the most derived subphyla so creating an argument in favour of a calcareous-shelled last common ancestor (LCA) of the Brachiopoda [65], or propose that phosphatic- and calcareous-shelled brachiopods are sister groups that had consistently distinct skeletal mineralogy from early in their phylogenetic history [66]. None of the known putative early Cambrian lophophorates have a skeleton suitable for a motile lifestyle but rather display evidence of a firm attachment [36,38,59–61]. In addition, both the earliest calcareous brachiopods (early Cambrian Obolellida) and the bryozoans (Ordovician Stenolaemata) possessed foliated ultrastructures [33,34].

## 7. Conclusion

We here suggest reinterpretation of the Ediacaran *Namacalathus* as sessile total group lophophorate. *Namacalathus* had an accretionary growth skeletal wall composed of a calcareous foliated ultrastructure together with columnar microlamellar inflections: features restricted to the Lophophorata only. *Namacalathus* additionally displays an internal organic-rich wall layer similar to that found in brachiopods and an internal budding being expressed in a symmetrical, bilateral pattern.

We conclude that at least some early lophophorates may have possessed a whole, rather than multicomponent sclerital, skeleton. These observations also suggest that the clonal, sessile and active suspension-feeding Lophophorata may have formed one of the earliest branches of the bilateria. Similarities in the overall biominerlization pattern between the Brachiopoda, the Bryozoa, the Tentaculitoidea and *Namacalathus* are likely due to a commonality in the molecular, cellular and physiological toolkits of biomineralization within a single group of related organisms inherited from their LCA.

## Supplementary Material

Supplementary Material

## Data accessibility

Additional methods and images supporting this article have been uploaded as part of the electronic supplementary material.

## References

[1] Erwin DH, Laflamme M, Tweedt SM, Sperling EA, Pisani D, Peterson KJ 2011 The Cambrian conundrum: early divergence and later ecological success in the early history of animals. Science 334, 1091–1097. (10.1126/science.1206375) 22116879

[2] Kouchinsky A, Bengtson S, Runnegar B, Skovsted C, Steiner M, Vendrasco M 2012 Chronology of early Cambrian biomineralization. Geol. Mag. 149, 221–251. (10.1017/S0016756811000720)

[3] Germs GJB 1972 New shelly fossils from the Nama Group, South West Africa. Am. J. Sci. 272, 752–761. (10.2475/ajs.272.8.752)

[4] Grotzinger JP, Watters WA, Knoll AH 2000 Calcified metazoans in thrombolite–stromatolite reefs of the terminal Proterozoic Nama Group, Namibia. Paleobiology 26, 334–359. (10.1666/0094-8373(2000)026<0334:CMITSR>2.0.CO;2)

[5] Wood RA, Grotzinger JP, Dickson JAD 2002 Proterozoic modular biomineralized metazoan from the Nama Group, Namibia. Sc*ience* 296, 2383–2386. (10.1126/science.1071599) 12089440

[6] Zhuravlev AYu, Liñán E, Gámez Vintaned JA, Debrenne F, Fedorov AB 2012 New finds of skeletal fossils in the terminal Neoproterozoic of the Siberian Platform and Spain. Acta Palaeontol. Pol. 57, 205–224. (10.4202/app.2010.0074)

[7] Cai Y, Xiao S, Hua H, Yuan X 2015 New material on the biomineralizing tubular fossil *Sinotubulites* from the late Ediacaran Dengying Formation, South China. Precambrian Res. 261, 12–24. (10.1016/j.precamres.2015.02.002)

[8] Kouchinsky A, Bengtson S 2002 The tube wall of Cambrian anabaritids. Acta Palaeontol. Pol. 47, 431–444. (http://www.paleo.pan.pl/acta/acta47/app47-431.pdf)

[9] Vinn O, Zaton M 2012 Inconsistencies in proposed annelid affinities of early biomineralized organism *Cloudina* (Ediacaran): structural and ontogenic features. Carnets Géol. Article 2012/03 (CG2012_A03), 39–47.

[10] Grotzinger JP, Bowring S, Saylor BZ, Kaufman AJ 1995 Biostratigraphic and geochronologic constraints on early animal evolution. Science 270, 598–604. (10.1126/science.270.5236.598)

[11] Hofmann HJ, Mountjoy EW 2001 *Namacalathus*–*Cloudina* assemblage in Neoproterozoic Miette Group (Byng Formation), British Columbia: Canada's oldest shelly fossils. Geology 29, 1091–1094. (10.1130/0091-7613(2001)029<1091:NCAINM>2.0.CO;2)

[12] Amthor JE, Grotzinger JP, Schröder S, Bowring SA, Ramezani J, Martin MW, Matter A 2003 Extinction of *Cloudina* and *Namacalathus* at the Precambrian–Cambrian boundary in Oman. Geology 31, 431–434. (10.1130/0091-7613(2003))

[13] Kontorovich AE et al. 2008 A section of Vendian in the east of West Siberian Plate (based on data from the Borehole Vostok 3). Russ. Geol. Geophys. 49, 932–939. (10.1016/j.rgg.2008.06.012)

[14] Terleev AA et al. 2011 *Cloudina–Namacalathus–Korilophyton* association in the Vendian of the Altay-Sayan Foldbelt (Siberia). In *Proc. of Int. Conf.**Neoproterozoic Sedimentary Basins: Stratigraphy*, *Geodynamics and Petroleum Potential*, Novosibirsk, 30 July–02 August 2011, pp. 96–98.

[15] Seilacher A, Grazhdankin D, Legouta A 2003 Ediacaran biota: the dawn of animal life in the shadow of giant protists. Paleontol. Res. 7, 43–54. (10.2517/prpsj.7.43)

[16] Wood RA 2011 Paleoecology of the earliest skeletal metazoan communities: implications for early biomineralization. Earth-Sci. Rev. 106, 184–190. (10.1016/j.earscirev.2011.01.011)

[17] Watters WA, Grotzinger JP 2001 Digital reconstruction of calcified early metazoans, terminal Proterozoic Nama Group, Namibia. Paleobiology 27, 159–171. (10.1666/0094-8373(2001)027<0159:DROCEM>2.0.CO;2)

[18] Zhuravlev AYu, Wood RA 2008 Eve of biomineralization: controls on skeletal mineralogy. Geology 36, 923–926. (10.1130/G25094A.1)

[19] Wood RA, Curtis A 2015 Extensive metazoan reefs from the Ediacaran Nama Group, Namibia: the rise of benthic suspension feeding. Geobiology 13, 112–122. (10.1111/gbi.12122) 25556318

[20] James NP, Klappa CF 1983 Petrogenesis of Early Cambrian reef limestones, Labrador, Canada. J. Sediment. Petrol. 53, 1051–1096.

[21] Wendt J 1979 Development of skeletal formation, microstructure, and mineralogy of rigid calcareous sponges from the Paleozoic to Recent. In Biologie des Spongiaires, Colloques Internationaux du C.N.R.S., Paris, vol. 291 (eds Lévi C, Boury-Esnault N), pp. 449–457.

[22] Wood RA 1987 Biology and revised systematics of some late Mesozoic stromatoporoids. Spec. Pap. Palaeontol. 37, 1–89.

[23] Alexander RR 1989 Influence of valve geometry, ornamentation, and microstructure on fractures in Late Ordovician brachiopods. Lethaia 22, 133–147. (10.1111/j.1502-3931.1989.tb01675.x)

[24] Vinn O, Mutvei H 2005 Observations on the morphology, and affinities of cornulitids from the Ordovician of Anticosti Island and the Silurian of Gotland. J. Paleontol. 79, 725–736. (10.1666/0022-3360(2005)079[0726:OOTMAA]2.0.CO;2)

[25] Connors MJ, Ehrlich H, Hog M, Godeffroy C, Araya S, Kallai I, Gazit D, Boyce M, Ortiz C 2012 Three-dimensional structure of the shell plate assembly of the chiton *Tonicella marmorea* and its biomechanical consequence. J. Struct. Biol. 177, 314–328. (10.1016/j.jsb.2011.12.019) 22248452

[26] Penny AM, Wood R, Curtis A, Bowyer F, Tostevin R, Hoffman K-H 2014 Ediacaran metazoan reefs from the Nama Group, Namibia. Science 344, 1504–1506. (10.1126/science.1253393) 24970084

[27] Taylor PD, Vinn O, Wilson MA 2010 Evolution of biomineralization in ‘lophophorates’. Spec. Pap. Palaeontol. 84, 317–333. (10.111/j.1475-4983.2010.00985.x)

[28] Williams A et al. 1997 Treatise on invertebrate paleontology, Pt H (Revised), Brachiopoda, *vol. 1* (ed. Kaesler RL). Boulder, CO: Geological Society of America; Lawrence, Kansas: University of Kansas Press.

[29] Williams A, Holmer LE 2002 Shell function and inferred growth functions and affinities of the sclerites of problematic *Micrina*. Palaeontology 45, 845–873. (10.1111/1475-4983.00264)

[30] Bidder GP 1923 The relation of the form of a sponge to its currents. Q. J. Microsc. Sci. 67, 293–323.

[31] Zhuravlev AYu 1993 A functional morphological approach to the biology of the Archaeocyatha. N. Jb. Geol. Paläont. Abh. 190, 315–327.

[32] Vinn O, Zaton M 2012 Phenetic phylogenetics of tentaculitoids—extinct, problematic tube-forming organisms. GFF 134, 145–156. (10.1080/11035897.2012.669788)

[33] Taylor PD, Lombardi C, Cocito S 2014 Biomineralization in bryozoans: present, past and future. Biol. Rev. Cambridge Philos. Soc. 90, 1118–1150. (10.1111/brv.12148) 25370313

[34] Ushatinsksaya GT, Malakhovskaya YaE 2006 First brachiopods with carbonate skeleton: appearance, migration, shell wall structure. In Evolution of the biosphere and the biodiversity (ed. Rozhnov SV), pp. 177–192. Moscow, Russia: Tovarishchestvo nauchnykh izdaniy KMK.

[35] Balthasar U 2008 *Mummpikia* gen. nov. and the origin of brachiopods. Palaeontology 51, 263–279. (10.1111/j.1475-4983.2008.00754.x)

[36] Skovsted CB, Holmer LE, Larsson CM, Hogstrom AES, Brock GA, Topper TP, Balthasar U, Stolk SP, Paterson JR 2009 The scleritome of *Paterimitra*: an early Cambrian stem group brachiopod from South Australia. Proc. R. Soc. B 276, 1651–1656. (10.1098/rspb.2008.1655) PMC2660981 19203919

[37] Larsson SM, Skovsted CB, Brock GA, Balthasar U, Topper TP, Holmer LE 2014 *Paterimitra pyramidalis* from South Australia: scleritome, shell structure and evolution of a lower Cambrian stem group brachiopod. Palaeontology 57, 417–446. (10.1111/pala.12072)

[38] Skovsted CB, Brock GA, Topper TP, Paterson JR, Holmer LE 2011 Scleritome construction, biofacies, biostratigraphy and systematics of the tommotiid *Eccentrotheca helenia* sp. nov. from the early Cambrian of South Australia. Palaeontology 54, 253–286. (10.1111/j.1475-4983.2010.01031.x)

[39] Zhuravlev AYu, Wood RA 2009 Controls on carbonate skeletal mineralogy: global CO_2_ evolution and mass extinctions. Geology 37, 1123–1126. (10.1130/G30204A.1)

[40] Bigatti G 2010 The calcareous egg capsule of the Patagonian neogastropod Odontocymbiola magellanica: morphology, secretion and mineralogy. J. Moll. Stud. 76, 279–288. (10.1093/mollusc/eyq006)

[41] Uthicke S, Mamigliano P, Fabricius KE 2013 High risk of extinction of benthic foraminifera in this century due to ocean acidification. Sci. Rep. 3, 1769 (10.1038/srep01769)

[42] Ippolitov AP, Vinn O, Kupriyanova EK, Jäger M 2014 Written in stone: history of serpulid polychaetes through time. Mem. Mus. Victoria 71, 123–159.

[43] Sköld HN, Obst M, Åkesson B 2009 Stem cells in asexual reproduction of marine invertebrates. In Stem cells in marine organisms (eds Rinkevich B, Matranga V), pp. 105–137. Dordrecht, The Netherlands: Springer Science+Business Media B.V.

[44] Banta WC 1972 The body wall of cheilostome Bryozoa, V. Frontal budding in *Schizoporella unicornis floridana*. Mat. Biol. 14, 63–71. (10.1007/bf00365783)

[45] Stancyk SE, Maturo FJS Jr, Heard RW Jr 1976 Phoronids from the east coast of the United States. Bull. Mar. Biol. 26, 576–584.

[46] Wilson MA, Vinn O, Yancey TE 2011 A new microconchid tubeworm from the Artinskian (Lower Permian) of central Texas, USA. Acta Palaeontol. Polonica 5, 785–791. (10.4202/app.2010.0086)

[47] Williams A, Holmer LE, Cusack M 2004 Chemico-structure of the organophosphatic shells of siphonotretide brachiopods. Palaeontology 47, 1313–1337. (10.1111/j.0031-0239.2004.00404.x)

[48] Jope HM 1965 Composition of brachiopod shell. In Treatise on invertebrate paleontology, Pt H, Brachiopoda (ed. Moore RC), pp. H159–H162. Boulder, CO: Geological Society of America; Lawrence, Kansas: University of Kansas Press.

[49] Williams A, Wright AD 1970 Shell structure of the Craniacea and other calcareous inarticulate Brachiopoda. Spec. Pap. Palaeontol. 7, 1–51.

[50] Kobayashi I, Samata T 2006 Bivalve shell structure and organic matrix. Mater. Sci. Eng. C 26, 692–698. (10.1016/j.msec.2005.09.101)

[51] Jackson DJ, Mann K, Haussermann V, Schilhabel MB, Luter C, Griesshaber E, Schmahl W, Worheide G 2015 The *Magellania venosa* biomineralizing proteome: a window into brachiopod shell evolution. Genome Biol. Evol. 7, 1349–1362. (10.1093/gbe/evv074) 25912046 PMC4453069

[52] Jackson DJ, Wörheide G 2014 Symbiophagy and biomineralization in the ‘living fossil’ *Astrosclera willeyana*. Autophagy 10, 408–415. (10.4161/auto.27319) 24343243 PMC4077880

[53] Helmkampf M, Bruchhaus I, Hausdorf B 2008 Phylogenetic analyses of lophophorates (brachiopods, phoronids and bryozoans) confirm the Lophotrochozoa concept. Proc. R. Soc. B 275, 1927–1933. (10.1098/rspb.2008.0372) PMC2593926 18495619

[54] Hausdorf B, Helmkampf M, Nesnidal M, Bruchhaus I 2010 Phylogenetic relationships within the lophophorate lineages (Ectoprocta, Brachiopoda and Phoronida). Mol. Phylogenet. Evol. 55, 1121–1127. (10.1016/j.ympev.2008.12.022) 20045074

[55] Nesnidal MP et al. 2013 New phylogenomic data support the monophyly of Lophophorata and an Ectoproct–Phoronid clade and indicate that Polyzoa and Kryptrochozoa are caused by systematic bias. BMC Evol. Biol. 13, 253 (10.1186/1471-2148-13-253) 24238092 PMC4225663

[56] Adrianov AV, Malakhov VV 2001 Symmetry of priapulids (Priapulida). 1. Symmetry of adults. J. Morphol. 247, 99–110. (10.1002/1097-4687(200102)247:2<99::AID-JMOR1005>3.0.CO;2-0) 11223921

[57] Steiner M, Qian Y, Li G, Hagadorn JW, Zhu M 2014 The developmental cycles of Early Cambrian Olivooidae fam. nov. (?Cycloneuralia) From the Yangtze Platform (China). Palaeogeogr. Palaeoclim. Palaeoecol. 398, 97–124. (10.1016/j.palaeo.2013.08.016)

[58] Dewel RA, Dewel WC, McKinney FK 2001 Diversification of the Metazoa: Ediacarans, colonies, and the origin of eumetazoan complexity by nested modularity. Hist. Biol. 15, 193–218. (10.1080/10292380109380592)

[59] Yang X, Vinn O, Hou X, Tian X 2013 New tubicolous problematic fossil with some ‘lophophorate’ affinities from the Early Cambrian Chengjiang biota in south China. GFF 135, 184–190. (10.1080/11035897.2013.801035)

[60] Zhang Z et al. 2013 A sclerite-bearing stem group ectoproct from the early Cambrian and its implications. Sci. Rep. 3, 1066 (10.1038/srep01066) 23336066 PMC3548229

[61] Zhang Z-F et al. 2014 An early Cambrian agglutinated tubular lophophorate with brachiopod characters. Sci. Rep. 4, 4682 (10.1038/srep04682) 24828016 PMC4021322

[62] Holmer LE, Skovsted CB, Williams A 2002 A stem group brachiopod from the Lower Cambrian: support for a *Micrina* (halkieriid) ancestry. Palaeontology 45, 875–882. (10.1111/1475-4983.00265)

[63] Temereva EN, Malakhov VV 2011 The evidence of metamery in adult brachiopods and phoronids. Invertebr. Zool. 8, 87–101.

[64] Fuchs J, Iseto T, Hirose M, Sundberg P, Obst M 2010 The first internal molecular phylogeny of the animal phylum Entoprocta (Kamptozoa). Mol. Phylogenet. Evol. 56, 370–379. (10.1016/j.ympev.2010.04.009) 20398775

[65] Carlson SJ 1995 Phylogenetic relationships among extant brachiopods. Cladistics 11, 131–197. (10.1111/j.1096-0031.1995.tb00084.x) 34920616

[66] Popov LE, Bassett MG, Holmer LE, Laurie J 1993 Phylogenetic analysis of higher taxa of Brachiopoda. Lethaia 26, 1–5. (10.1111/j.1502-3931.1993.tb01502.x)

