# Supplementary material for: Ediacaran skeletal metazoan interpreted as a lophophorate
Source: Proc Biol Sci. 2015 Nov 7;282(1818):20151860. doi: 10.1098/rspb.2015.1860 (PMC4650157; doi:10.1098/rspb.2015.1860)
Supplement: Supplementary Material [file rspb20151860supp1.doc]

**Supplementary Materials**

**Ediacaran skeletal metazoan interpreted as a lophophorate**

Zhuravlev, A. Yu., Wood, R. A., and Penny, A. M.

**3D reconstruction methodology**

Serial grinding was adopted as an imaging method due to the difficulty of distinguishing fossil from matrix in this material using available non-destructive 3D reconstruction methods such as computed tomography (CT) scanning. The fossils are preserved as calcite within a carbonate matrix, so there is no differences in x-ray density to distinguish fossil from matrix.

Two samples were serially ground by hand. The corners of the cut samples were used as registration points for aligning the images into a stack. Grinding was undertaken on two rotating grinding disks, with the first disk at 74µm grade to grind the sample down, followed by a 20µm grade disk to polish the ground surface and clarify features for scanning. Each grinding stage removed a sample thickness of approximately 100µm, measured after each grinding stage with a Mitutoyo micrometer screw gauge.

After each grinding stage, the resulting surface was washed to remove rock powder, and imaged wet at 1200dpi using a Canon CanoScan 9000F Mark II document scanner. The resulting series of images was used to generate 3D reconstructions using the software SPIERS (Sutton et al., 2012). The stack was imported into SPIERSAlign to be rotated into alignment and cropped to a uniform size before the image stack was imported into SPIERSEdit. Threshholding was then used to differentiate the darker skeletal material from the lighter matrix. After thresholding, some manual editing was required, where possible, to remove darker areas of matrix from the reconstruction. A 3D reconstruction was then generated in SPIERSView.


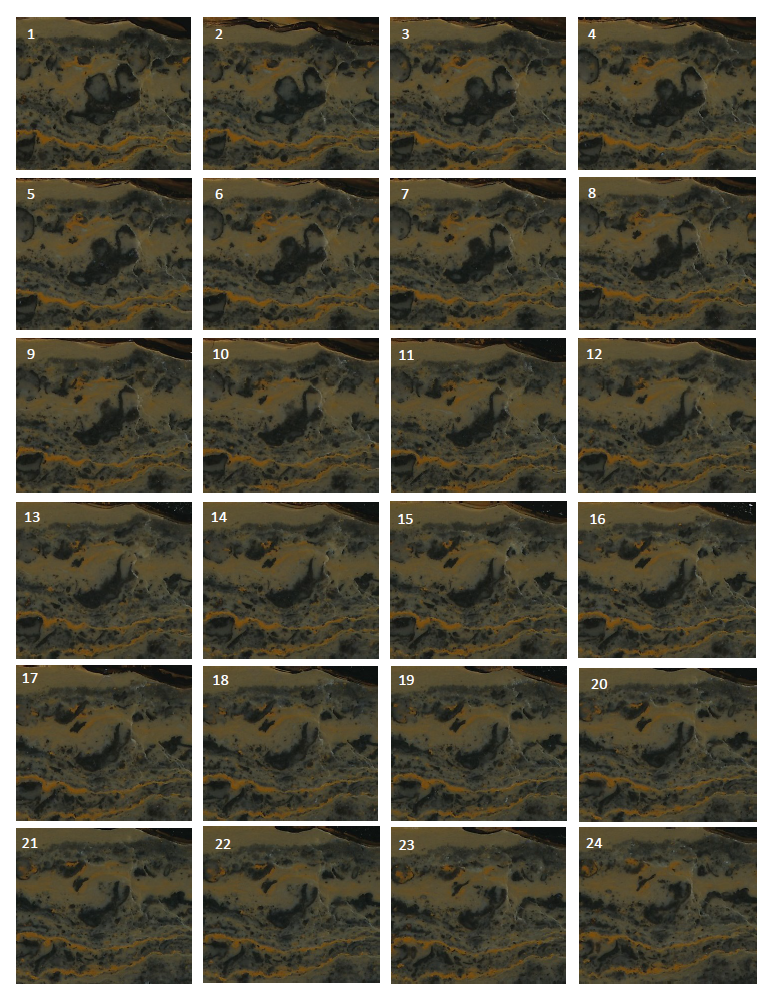


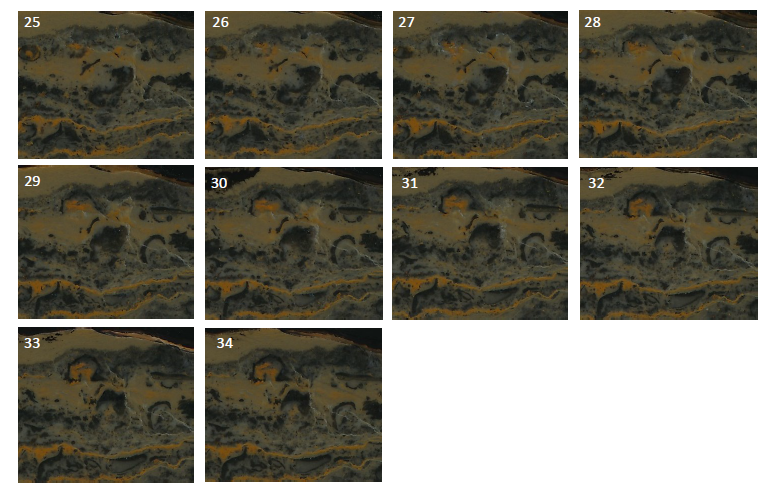


Fig. S1. Succesive seriel images 100 μm apart from sample shown in Fig. 1M. Width of images = 40 mm.


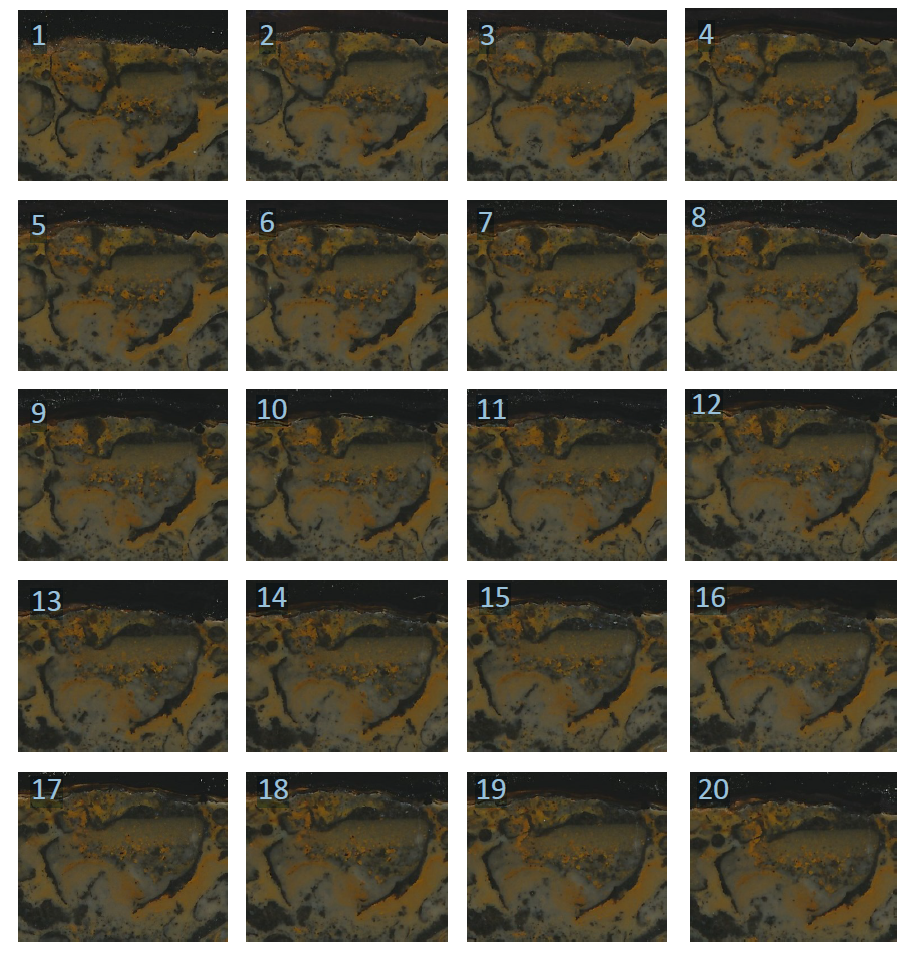


Fig. S2. Succesive seriel images 100 mm apart from sample shown in Fig. 1L. Width of images = 20 mm.


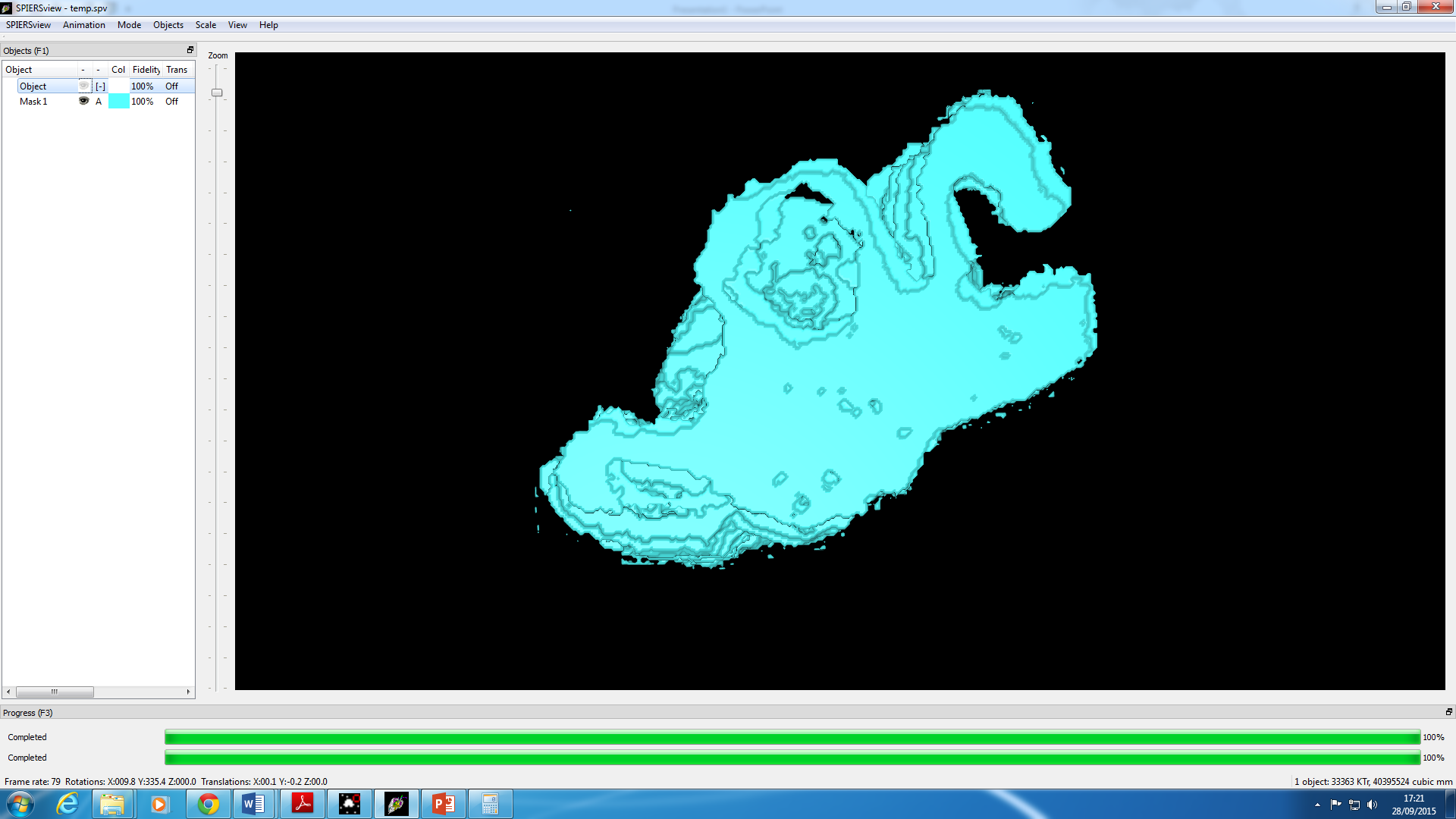

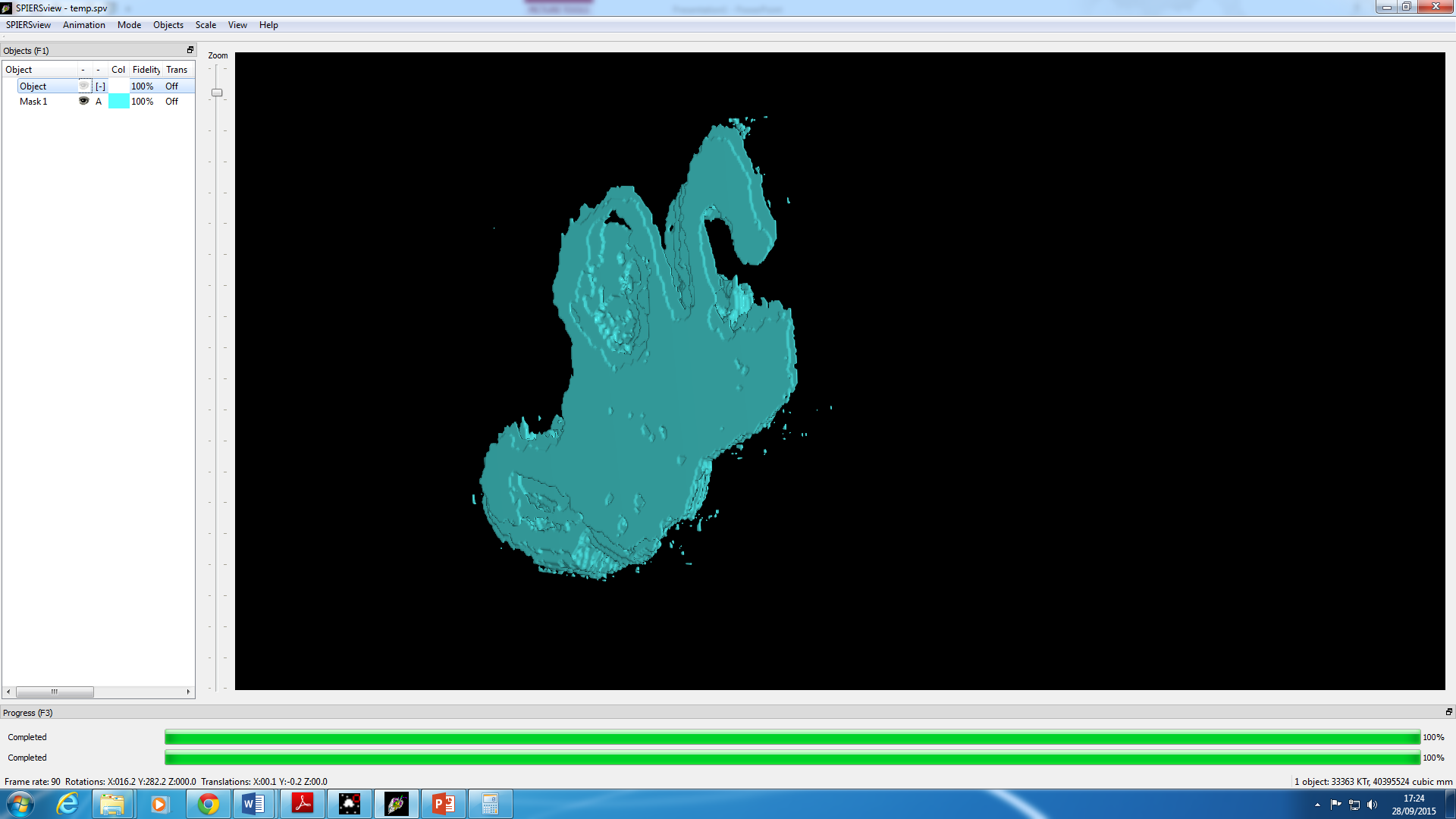

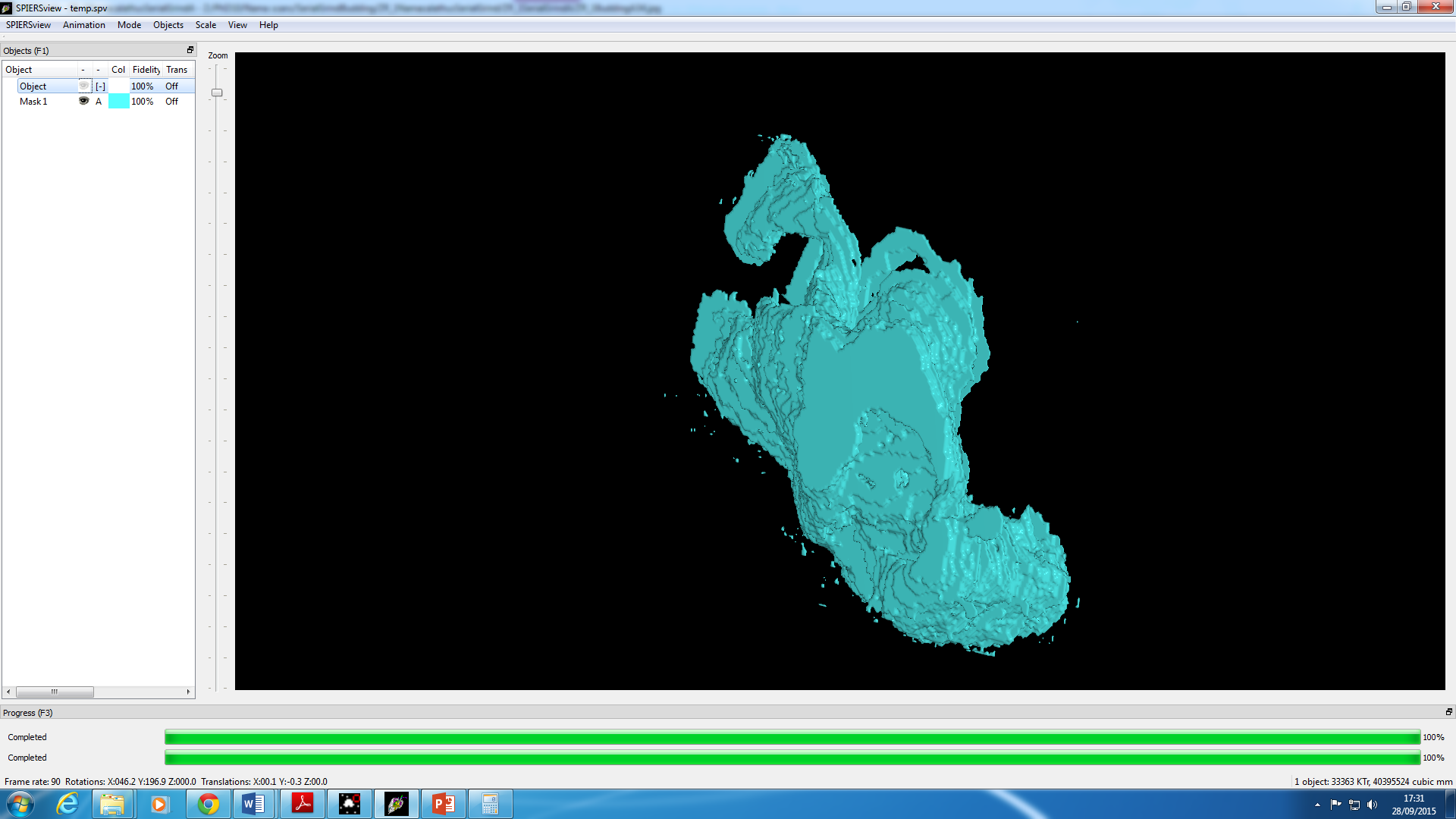

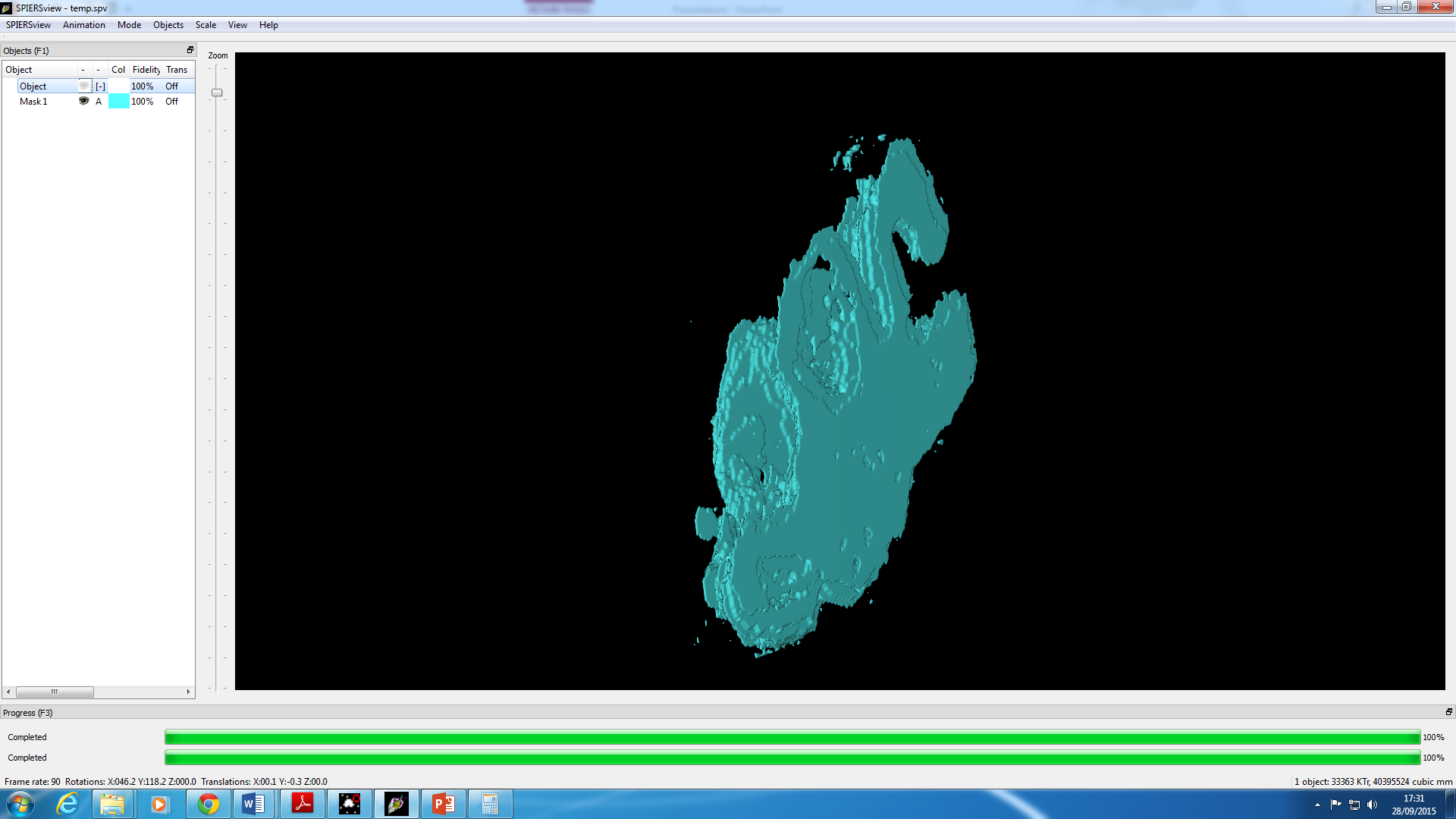


Fig. S3. Views of 3D model generated in SPIERS from sample shown in Fig. 1M.

Width of images = 25 mm.


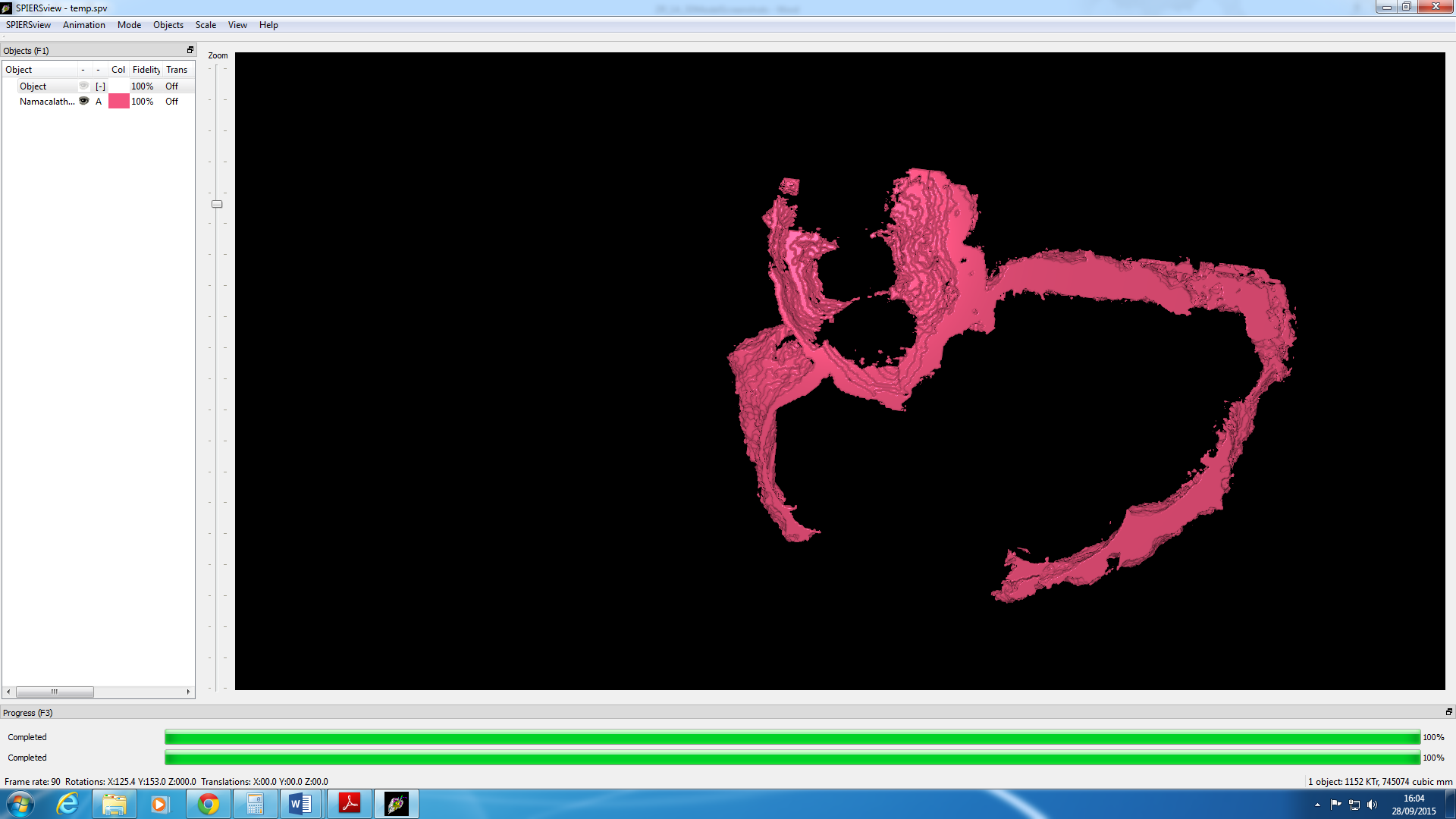

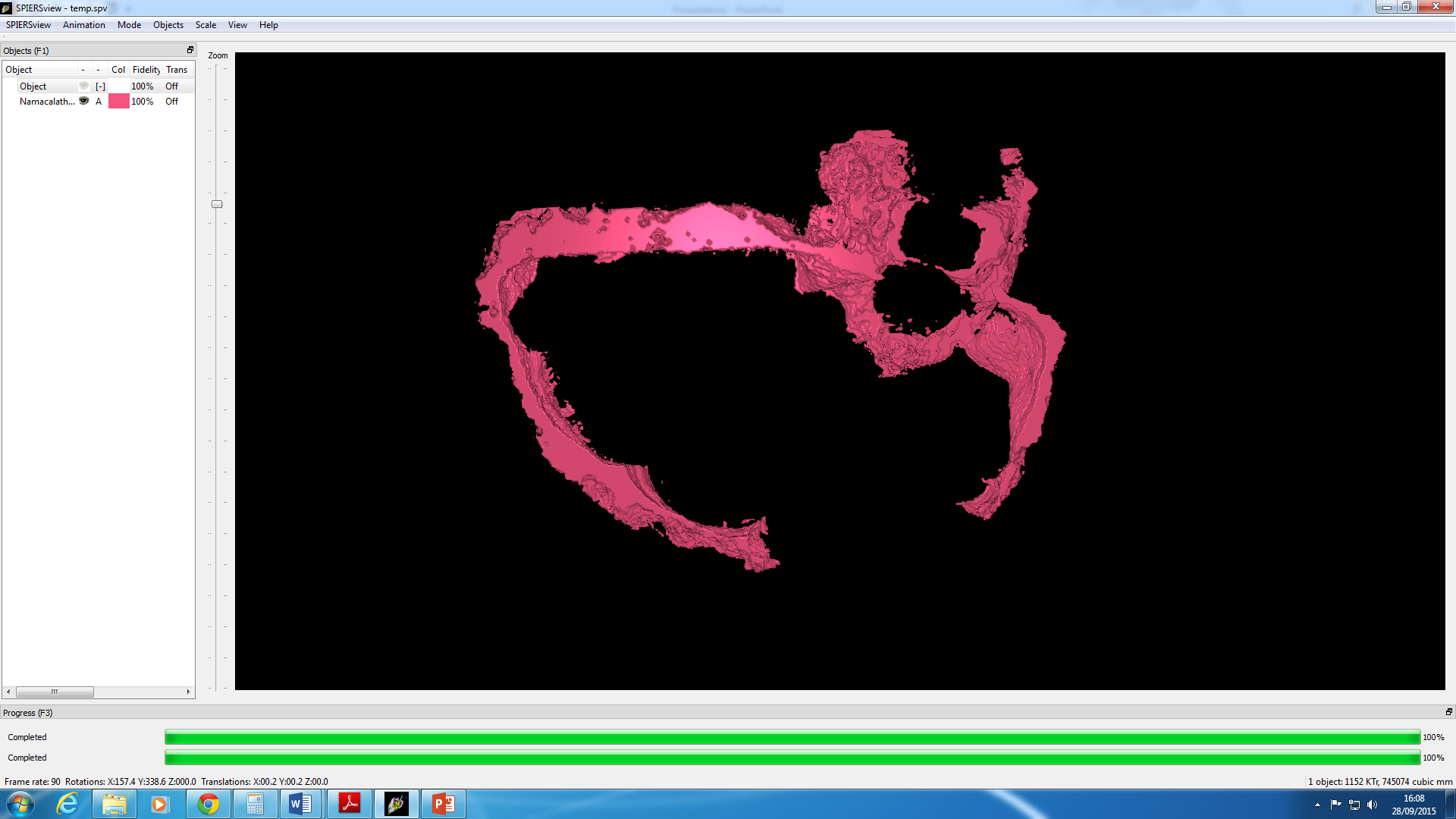

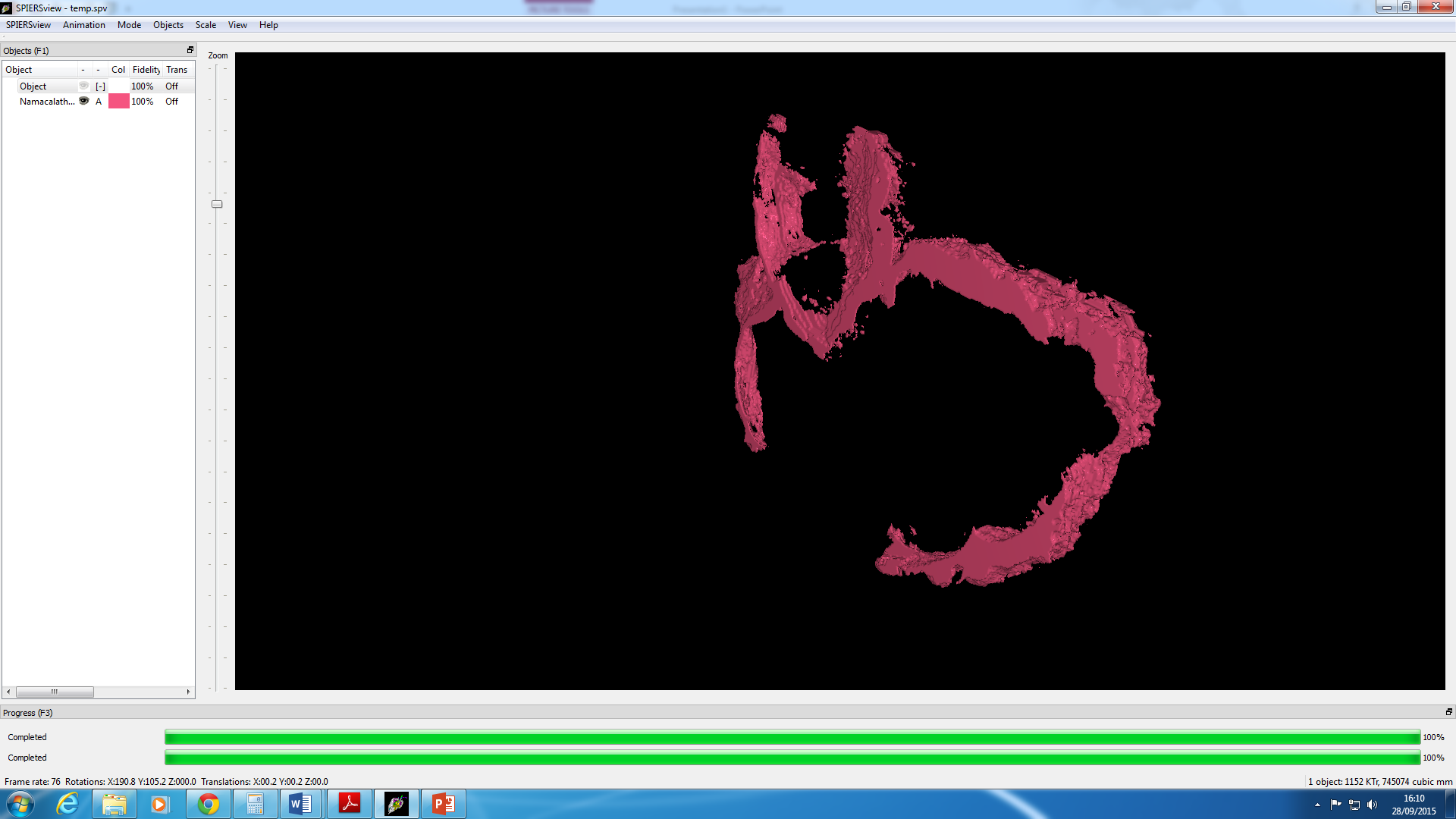

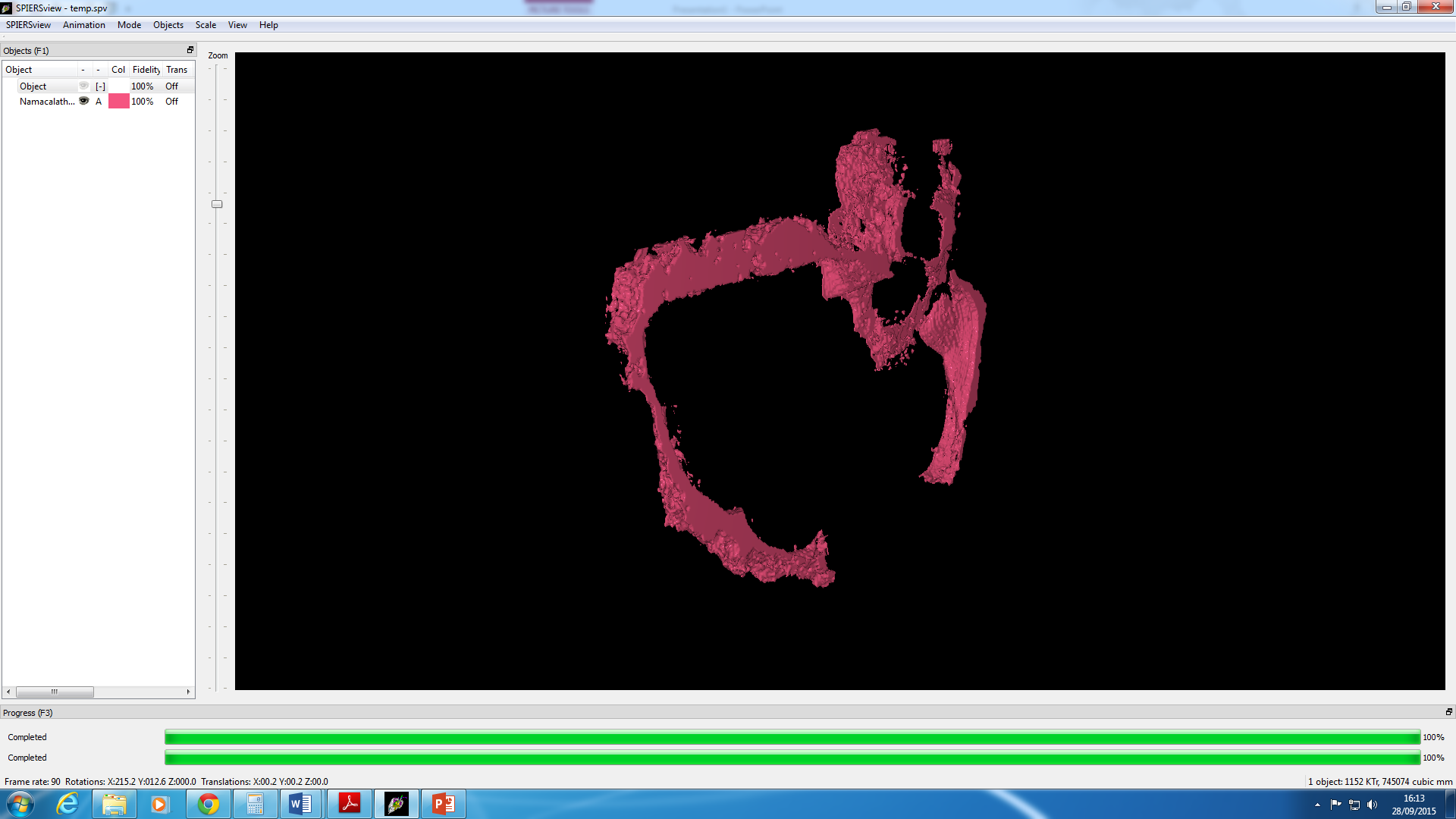

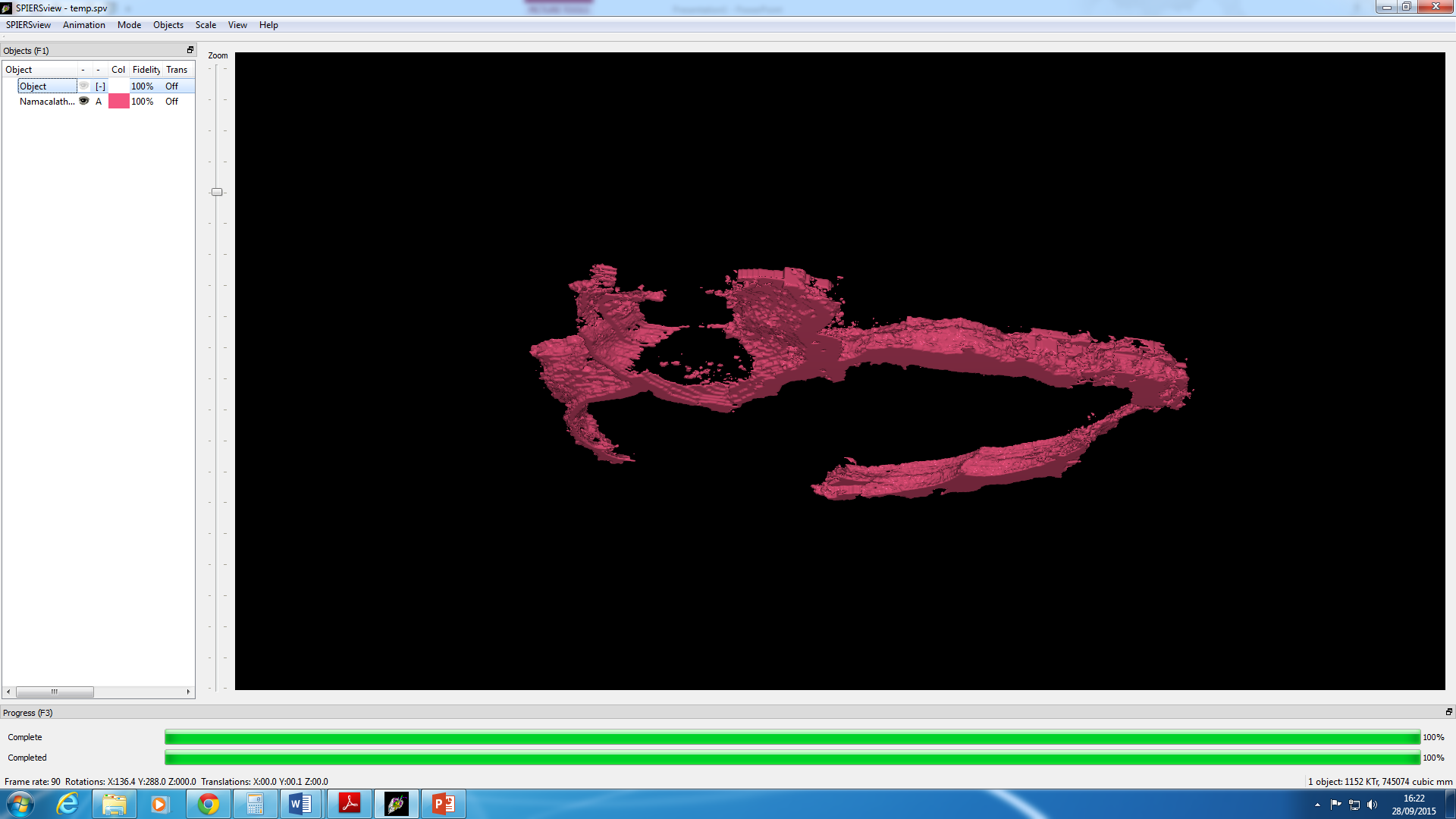

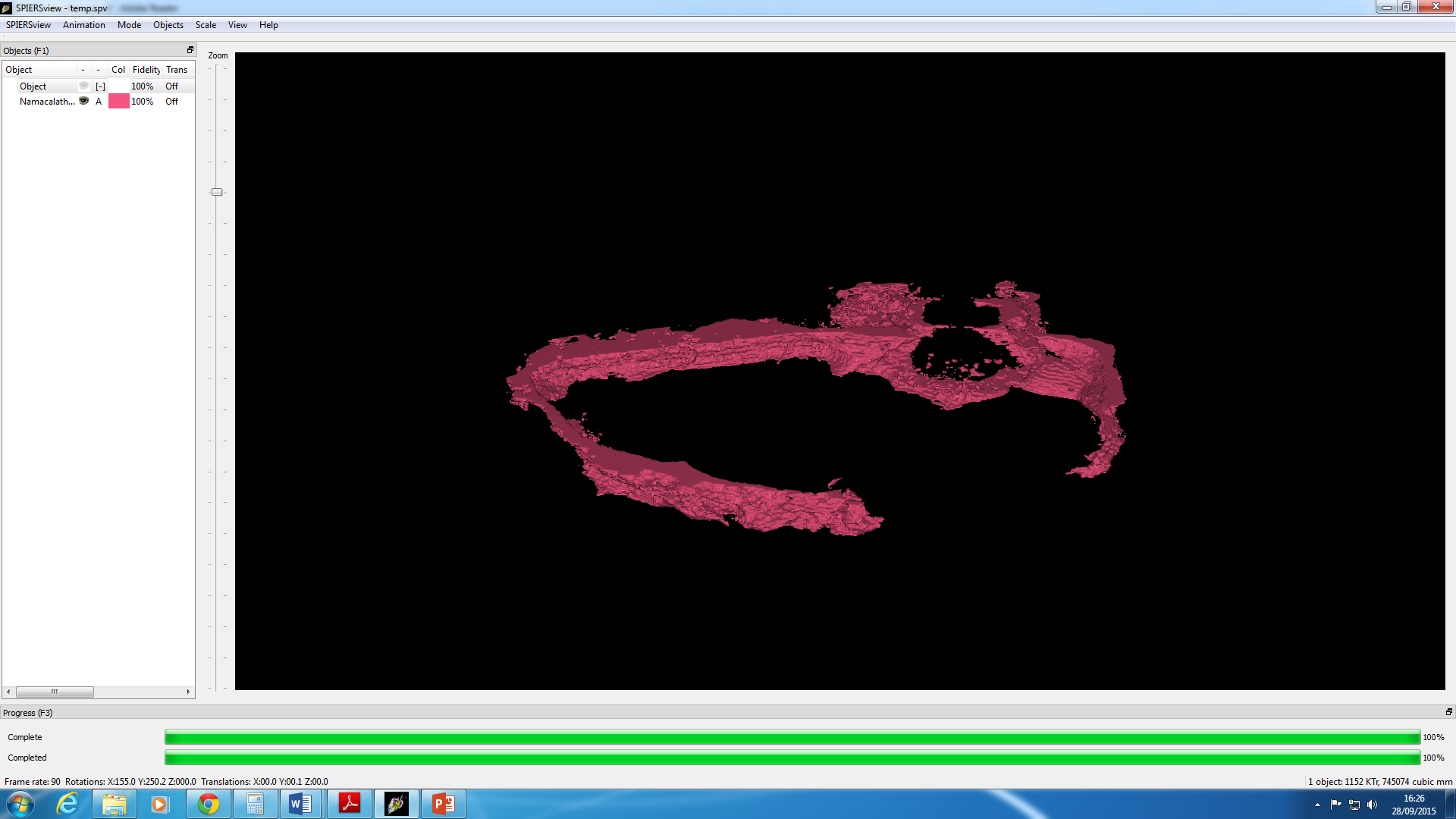


Fig. S4. Views of 3D model generated in SPIERS from sample shown in Fig. 1L.

Width of images = 20 mm.

Sutton, Mark D., Garwood, Russell J., Siveter, David J., Siveter, Derek J., 2012. 'SPIERS and VAXML; A software toolkit for tomographic visualisation and a format for virtual specimen interchange', Palaeontologia Electronica 15.2.5T: 15p
